# UTILITY OF THE REOPENABLE CLIP-OVER-THE-LINE METHOD FOR DEFECT CLOSURE AFTER ENDOSCOPIC INTERMUSCULAR DISSECTION OF RECTAL DEEP SUBMUCOSAL INVASIVE CANCER

**DOI:** 10.1590/S0004-2803.24612025-116

**Published:** 2026-03-23

**Authors:** Taiji YOSHIMOTO, Hiroo ISHII, Hiroshi Takihara

**Affiliations:** 1 Tachikawa Sogo Hospital, Department of Gastroenterology, Tokyo, Japan.; 2 Uji Tokushukai Hospital, Department of Gastroenterology, Uji, Kyoto, Japan.

## Case presentation

Endoscopic intermuscular dissection (EID) is a surgical technique developed to achieve free vertical margins in the treatment of rectal deep submucosal invasive cancer ^1^ ^,^ ^2^. During EID, the inner circular muscle layer is dissected. Because EID carries a higher risk of postoperative complications, such as bleeding and perforation, than endoscopic submucosal dissection (ESD), prophylactic defect closure should be considered to ensure safety. EID is mainly performed in the rectum, where the thicker wall makes defect closure more technically challenging than in the colon.

**FIGURE 1:**
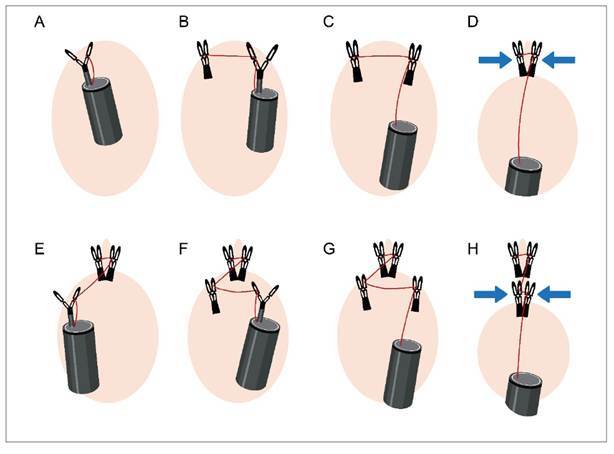
Schematic diagram of the reopenable clip-over-the-line method (ROLM) of defect closure. **(A)** A clip is tied with thread and inserted into the accessory channel of the endoscope, and the defect edge is grasped; **(B, C)** The thread is passed through a hole in one of the teeth of the second clip. The contralateral edge of the defect is then grasped using the second clip; **(D)** The defect is approximated by pulling the thread through (blue arrows); **(E, F, G)** A third threaded clip is placed on the contralateral edge of the defect, followed by a fourth threaded clip on the opposite defect edge; **(H)** Further approximation is achieved by pulling the thread through (blue arrows). This procedure is repeated until the defect is completely closed.

The reopenable clip-over-the-line method (ROLM) for defect closure uses a clip that can be repeatedly opened and closed (Sure Clip; Micro-Tech Co., Ltd., Nanjing, China) and a nylon thread^3^ ^-^ ^6^. First, the thread is tied to the clip, and the clip is inserted into the accessory channel of the endoscope. The edge of the defect is then grasped using the clip. Next, the thread is passed through the hole in one of the teeth of the second clip. The contralateral edge of the defect is grasped with the second clip, and the thread is pulled through. A third threaded clip is subsequently placed on the opposite side of the defect, and the thread is again pulled through. This sequence is repeated until complete closure of the defect is achieved (Figure 1). Finally, the thread is cut using scissor forceps.

**FIGURE 2:**
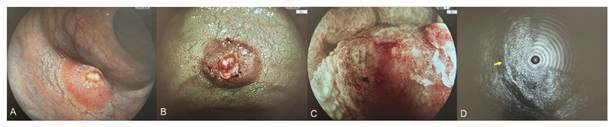
Rectal deep submucosal invasive cancer in a 78-year-old man. **(A)** the lesion, located in the lower rectum, was a Paris classification macroscopic type 0-Is + IIc lesion measuring approximately 10 mm in diameter; **(B, C)** narrow-band imaging (NBI) showed a type 3 pattern according to the Japan NBI Expert Team classification system; **(D)** endoscopic ultrasonography using a mini-probe revealed involvement of the submucosal layer without invasion of the muscularis propria (yellow arrow).

We present the case of a 78-year-old man with a type 0-Is + IIc (Paris classification) rectal lesion measuring approximately 10 mm in diameter. Narrow-band imaging (NBI) identified the lesion as type 3 according to the Japan NBI Expert Team classification system. Endoscopic ultrasonography using a mini-probe revealed involvement of the submucosal layer without invasion of the muscularis propria (Figure 2). Based on these findings, the patient was scheduled for tumor resection. EID was performed, resulting in en bloc resection without adverse events or complications. The ROLM was then applied for defect closure, achieving complete closure of the defect with no residual submucosal dead space (Figure 3 and E-VIDEO). 

**FIGURE 3:**
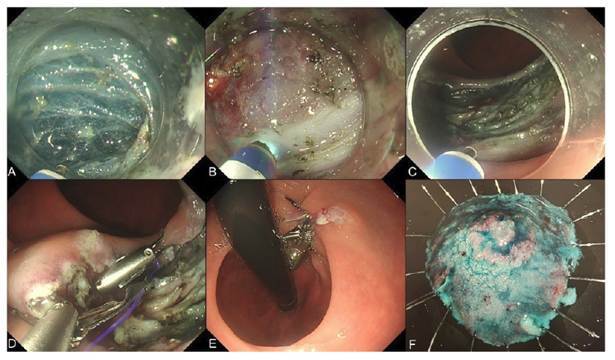
Endoscopic images of the reopenable clip-over-the-line method (ROLM) used for defect closure after endoscopic intermuscular dissection (EID). **(A)** creation of a submucosal tunnel on both sides of the lesion; **(B)** dissection of the inner circular muscle layer; **(C)** mucosal defect following EID; **(D)** defect closure using the ROLM; (E) complete closure of the mucosal defect; (F) the resected specimen.

The ROLM offers several advantages over other defect closure techniques. First, suturing can be easily achieved by grasping the defect edge and pulling the thread. Second, neither special devices nor a large working space for endoscope manipulation are required. Finally, endoscope withdrawal is unnecessary, and there is no limitation on the size of the defect that can be closed. At our institution, the ROLM has been used for defect closure in multiple cases of rectal EID, and complete closure was consistently achieved.

In conclusion, the ROLM is an effective and practical method for defect closure following EID.

## Video Caption

Utility of the reopenable clip-over-the-line method for defect closure after endoscopic intermuscular dissection of rectal deep submucosal invasive cancer.

## Data Availability

data in article: the research data are presented within the article itself (including the case description, Figures 1-3, and the accompanying video).

## References

[1] Rahni DO, Toyonaga T, Ohara Y, Lombardo F, Baba S, Takihara H (2017). First reported case of per anal endoscopic myectomy (PAEM): a
novel endoscopic technique for resection of lesions with severe fibrosis in
the rectum. Endosc Int Open.

[2] Moons LMG, Bastiaansen BAJ, Richir MC, Hazen WL, Tuynman J, Elias SG (2022). Endoscopic intermuscular dissection for deep submucosal invasive
cancer in the rectum: a new endoscopic approach. Endoscopy.

[3] Nomura T, Sugimoto S, Tsuda N, Matsushima R, Oyamada J, Kamei A (2021). Mucosal defect closure after duodenal endoscopic submucosal
dissection using the reopenable-clip over the line method. JGH Open.

[4] Nomura T, Sugimoto S, Temma T, Oyamada J, Ito K, Kamei A (2023). Reopenable clip-over-the-line method for closing large mucosal
defects following colorectal endoscopic submucosal dissection: a feasibility
study. Endosc Int Open.

[5] Shichijo S, Uedo N, Mori H, Kawakami Y, Tani Y, Iwagami H (2025). Reopenable clip over-the-line method in endoscopic full-thickness
resection of gastric submucosal tumors: a historical control
study. DEN Open.

[6] Sugimoto S, Nomura T, Temma T, Sawa E, Omae K, Tsuda N (2025). Closure of gastric mucosal defects using the reopenable-clip
over-the-line method to decrease the risk of bleeding after endoscopic
submucosal dissection: a multicenter propensity score-matched case-control
study (with video). Gastrointest Endosc.

